# Intra- and Inter-Tumor *BRAF* Heterogeneity in Acral Melanoma: An Immunohistochemical Analysis

**DOI:** 10.3390/ijms20246191

**Published:** 2019-12-08

**Authors:** Takamichi Ito, Yumiko Kaku-Ito, Maho Murata, Toshio Ichiki, Yuki Kuma, Yuka Tanaka, Taketoshi Ide, Fumitaka Ohno, Maiko Wada-Ohno, Yuichi Yamada, Yoshinao Oda, Masutaka Furue

**Affiliations:** 1 Department of Dermatology, Graduate School of Medical Sciences, Kyushu University, Fukuoka 812-8582, Japan; kyumiko@dermatol.med.kyushu-u.ac.jp (Y.K.-I.); muratama@dermatol.med.kyushu-u.ac.jp (M.M.); itoshio@dermatol.med.kyushu-u.ac.jp (T.I.); yukikuma@dermatol.med.kyushu-u.ac.jp (Y.K.); yukat53@med.kyushu-u.ac.jp (Y.T.); taketosi@dermatol.med.kyushu-u.ac.jp (T.I.); fumi16@dermatol.med.kyushu-u.ac.jp (F.O.); maiko@dermatol.med.kyushu-u.ac.jp (M.W.-O.); furue@dermatol.med.kyushu-u.ac.jp (M.F.); 2 Department of Anatomic Pathology, Graduate School of Medical Sciences, Kyushu University, Fukuoka 812-8582, Japan; yyamada@surgpath.med.kyushu-u.ac.jp (Y.Y.); oda@surgpath.med.kyushu-u.ac.jp (Y.O.)

**Keywords:** acral melanoma, *BRAF*, gene, genetics, heterogeneity, immunohistochemistry, mutation

## Abstract

The current development of BRAF inhibitors has revolutionized the treatment of unresectable melanoma. As the potential heterogeneity of *BRAF* mutations in melanoma has been reported, accurate detection of *BRAF* mutations are important. However, the genetic heterogeneity of acral melanoma—a distinct type of melanoma with a unique genetic background—has not fully been investigated. We conducted a retrospective review of our acral melanoma patients. Of the 196 patients with acral melanoma, we retrieved 31 pairs of primary and matched metastatic melanomas. We immunostained the 31 pairs with VE1, a *BRAF*^V600E^-mutation-specific monoclonal antibody. Immunohistochemistry with VE1 showed a high degree of sensitivity and specificity for detecting *BRAF*^V600E^ mutations compared with the real-time polymerase chain reaction method. A total of nine primary (29.0%) and eight metastatic (25.8%) acral melanomas were positive for VE1. In three patients (9.7%), we observed a discordance of VE1 staining between the primary and metastatic lesions. Of note, VE1 immunohistochemical staining revealed a remarkable degree of intra-tumor genetic heterogeneity in acral melanoma. Our study reveals that VE1 immunostaining is a useful ancillary method for detecting *BRAF*^V600E^ mutations in acral melanoma and allows for a clear visualization of intra- and inter-tumor *BRAF* heterogeneity.

## 1. Introduction

Malignant melanoma is an aggressive malignant tumor derived from melanocytes, with increasing cases worldwide [1,2]. Malignant melanoma can occur all over the body but is found most frequently on the skin. Early-stage melanoma can be cured with simple surgical removal, whereas advanced or metastatic melanomas are life threatening. Recent developments in immunotherapy and molecular targeted therapy have revolutionized the treatment of metastatic melanoma. Compared with conventional chemotherapy with dacarbazine, they have improved the rates of overall and progression-free survival [3,4,5,6].

Approximately half of cutaneous melanomas harbor driver gene mutations, such as *BRAF* mutations, depending on the type of melanoma and the patient’s degree of sunlight exposure. The *BRAF* mutation constitutively activates BRAF and its downstream mitogen-activated protein-kinase (MAPK) pathway, leading to the aberrant proliferation and survival of melanoma cells [7,8]. BRAF/MEK inhibitors, such as vemurafenib/cobimetinib and dabrafenib/trametinib, target melanoma cells with oncogenic *BRAF* mutations, including *BRAF*^V600E^ and *BRAF*^V600K^. The latest version of the National Comprehensive Cancer Network (NCCN) guidelines recommends BRAF inhibitors in combination with MEK inhibitors as one of the first-line treatments for unresectable melanoma with *BRAF* mutations [9]. In clinical settings, whether a melanoma has *BRAF* mutations is a major concern, because the patients’ *BRAF* status influences the treatment strategy.

Multiple molecular techniques have been used to detect *BRAF* mutations, including allele-specific real-time PCR (RT-PCR), high-resolution melting, Sanger sequencing, and pyrosequencing [10]. Cobas BRAF V600E Mutation Test (Roche Diagnostics, Mannheim, Germany), an approved, commercially available test, which is widely used in many countries, performs real-time PCR analysis and can detect *BRAF* mutant alleles with a limit of 5% [11]. In addition to these molecular detection techniques, immunohistochemistry (IHC) using the VE1 antibody may be an alternative in *BRAF* mutation screening, as it offers highly concordant results regarding mutation status based on molecular analysis [12,13].

As in many other tumors, molecular heterogeneity is reported in melanoma [14,15,16]. Tumor heterogeneity refers to the existence of subpopulations of cells with distinct molecular variation within individual tumors (intra-tumor heterogeneity) or between tumors at different sites within a patient (inter-tumor heterogeneity). Previous studies have revealed that melanoma is associated with inter-tumor heterogenous *BRAF* status in about 4%–25% of patients [17,18]. If wild-type *BRAF* melanoma cells are exposed to BRAF inhibitors, the melanoma cells may paradoxically activate the MAPK pathway [18], causing adverse effects. Therefore, accurate detection of *BRAF* status and possible genetic heterogeneity is important in managing melanoma.

Acral melanoma is a distinct subtype of melanoma that arises on the palms, on the soles of the feet, and under the nails [19,20,21]. Acral melanoma characteristically has a different genetic background compared with other subtypes (e.g., infrequent *BRAF* mutations and frequent *KIT* mutations) [22], and mechanical stress (rather than ultraviolet irradiation) may be a causative factor [23]. Although acral melanoma accounts for <10% of all melanoma in Caucasian populations, the proportion of acral melanoma is much higher in Asian populations and individuals with dark skin [19,20,21]. Unfortunately, the genetic heterogeneities of acral melanomas have rarely been investigated.

In this study, we sought to determine the *BRAF*^V600E^ mutation status in primary and metastatic acral melanoma patients and to assess the consistency between the results of IHC and molecular analysis. We also retrospectively investigated *BRAF*^V600E^ mutational heterogeneity among different subclones within an individual tumor (intra-tumor heterogeneity) and between primary and metastatic melanoma within a patient (inter-tumor heterogeneity) using 31 pairs of primary and metastatic acral melanoma samples from our patients.

## 2. Results

### 2.1. Patient Data

Of the 196 patients with acral melanoma treated at our hospital between June 2001 and June 2019, we identified a total of 31 patients whose primary and metastatic tissues were available. Table 1 shows the clinicopathological data of all 31 patients. All the patients were Japanese; 15 patients (48.4%) were male, and 16 patients (51.4%) were female; the mean age was 69.8 years. Among our patients, the primary tumor site was predominantly on the foot (74.2%), and metastases were mainly on the lymph nodes (77.4%), followed by the skin (19.4%) and the lungs (3.3%).

### 2.2. Consistency between IHC and RT-PCR in BRAF^V600E^ Status in Acral Melanoma

Among the 31 patients, *BRAF*^V600E^ status was established in 10 patients using the FDA-approved companion diagnostic kit Cobas BRAF V600 Mutation Test (Roche Diagnostics). Initially, we examined the consistency in reported *BRAF*^V600E^ status, comparing IHC results to those tested using the companion kit. To detect the mutated BRAF-V600E protein, we used a mouse monoclonal antibody (VE1, ab228461; Abcam, Cambridge, UK) as the primary antibody. As a chromogen, we used FastRed II (Nichirei Biosciences, Tokyo, Japan), which shows positive signals in red, enabling researchers to easily distinguish positive signals (in red) from brown melanin. Table 2 summarizes the *BRAF*^V600E^ status of the 10 patients. One patient had VE1-positive melanoma, whereas the remaining nine had negative results following IHC, which was in close accordance with the RT–PCR results. The sensitivity and specificity of IHC were 100% and 100%, respectively.

### 2.3. Results of IHC Staining

**Figure 1:**
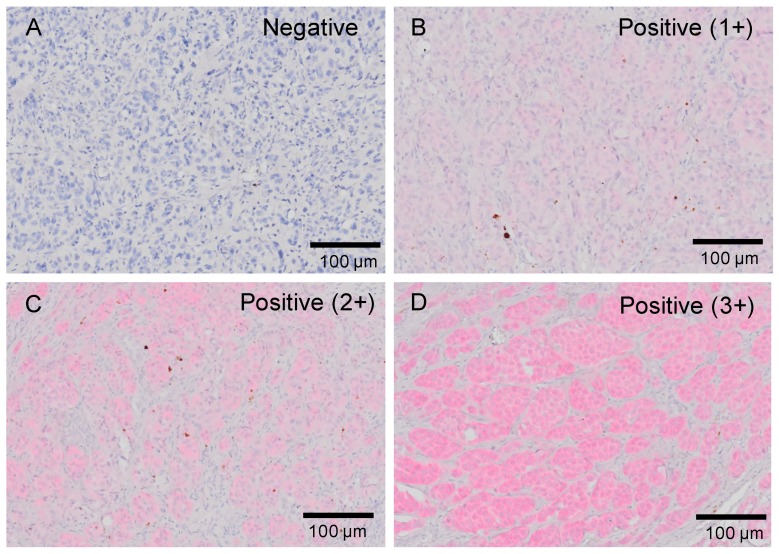
Staining intensity of VE1 antibody. Positive signals are expressed in red. (**A**) Negative staining. (**B**) Mildly positive staining (1+). (**C**) Moderately positive staining (2+). (**D**) Strongly positive staining (3+). Bars, 100 μm.

Because the staining of VE1 in acral melanoma varied in both intensity and proportion, we regarded tissues as mutation-positive when at least 5% of melanoma cells expressed a positive staining of 1–3+, in accordance with the instructions of the Cobas BRAF V600E Mutation Test, which indicated a cutoff value of >5% of tumor DNA. The intensity of staining was judged on a semiquantitative scale of 0–3+: no staining (0), weakly positive staining (1+), moderately positive staining (2+), and strongly positive staining (3+). Each slide was independently assessed by two dermatopathologists (T.I. and Y.K-I.), who were blinded to patients’ clinicopathological and mutation data. Slides that received different evaluations were reviewed by both observers together, and discrepancies were resolved by consensus. Figure 1 shows representative images of VE1 staining and intensity scores. Intensities of ≥1+ cytoplasmic staining were defined as positive. Heterogenous intensity was defined as, distinct subpopulations of melanoma cells with both positive and negative immunoreactivities.

**Figure 2:**
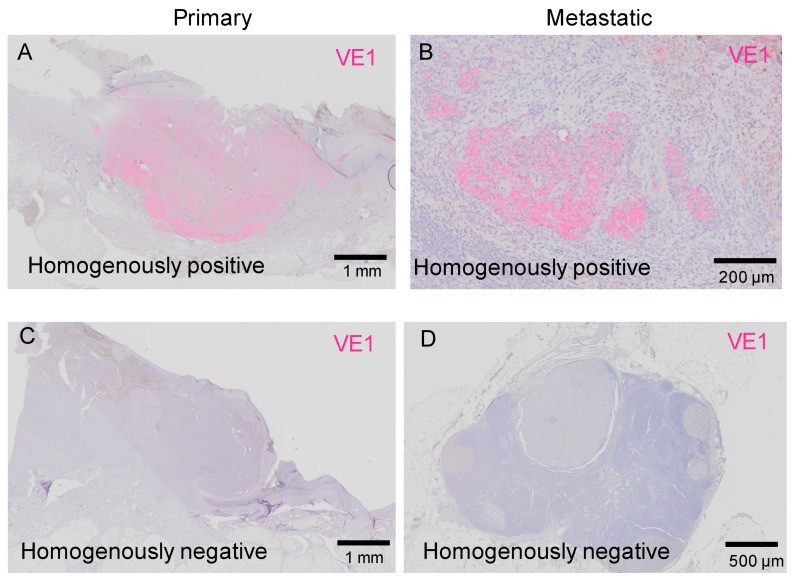
Representative cases of homogeneous staining of VE1 antibody. (**A**,**B**) Homogenously positive staining of primary (**A**) and metastatic (**B**) acral melanoma in a single patient. (**C**,**D**) Homogenously negative staining of primary (**C**) and metastatic (**D**) acral melanoma in another patient. Bars indicate 1 mm in (**A,C**), 200 μm in (**B**), and 500 μm in (**D**).

Figure 2 shows cases of homogenous staining of VE1. Only a few melanoma cells showed homogenous VE1 positivity (Figure 2A,B), while most of the melanomas showed homogenous VE1 negativity (Figure 2C,D). The tumors in Figure 2A,B were from the same patient, thus showing concordant *BRAF*^V600E^ status between primary and metastatic tumors. Similarly, the tumors in Figure 2C,D were both taken from a different patient in the study group.

#### 2.3.1. Intra-Tumor Heterogeneity in *BRAF*^V600E^ Status in Acral Melanoma

**Figure 3:**
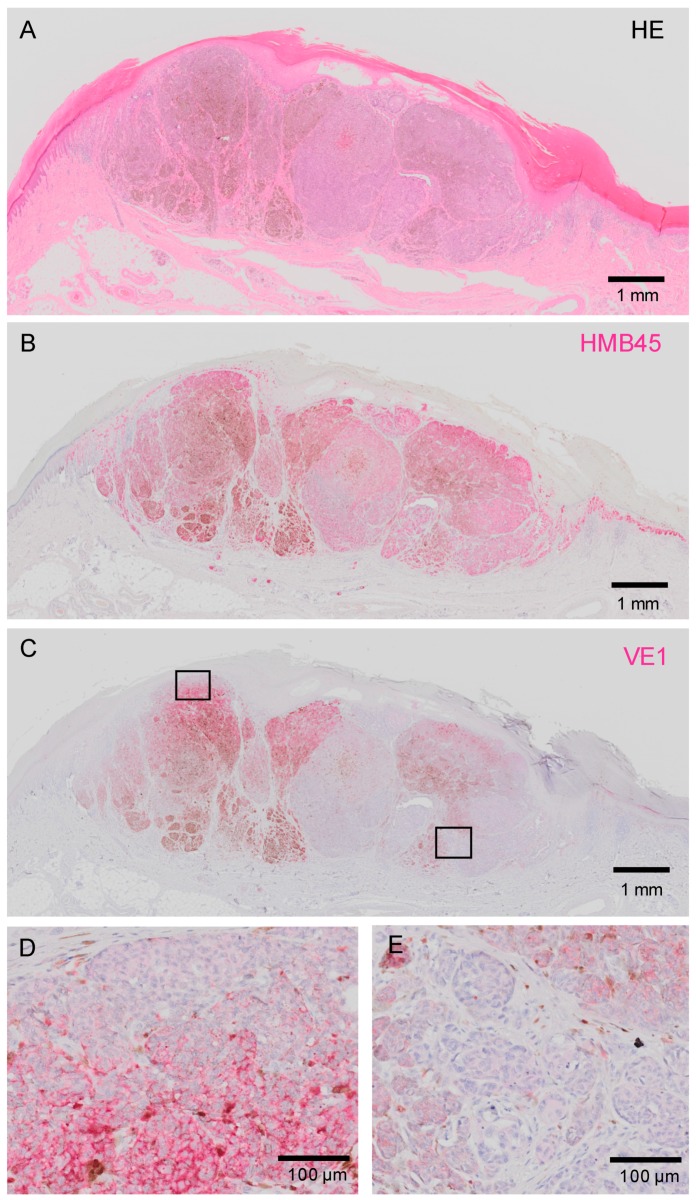
A representative case of acral melanoma. (**A**,**B**) HMB45 clearly highlights the melanoma cells in red. (**C**) Low-power view of VE1 staining. A highly heterogenous staining is evident. (**D**,**E**) High-power view of areas showing strong (**D**) and negative to weak (**E**) staining as indicated by rectangles in (**C**). Heterogenous staining is also evident. Bars indicate 1 mm in (**A**–**C**) and 100 μm in (**D**,**E**).

**Figure 4:**
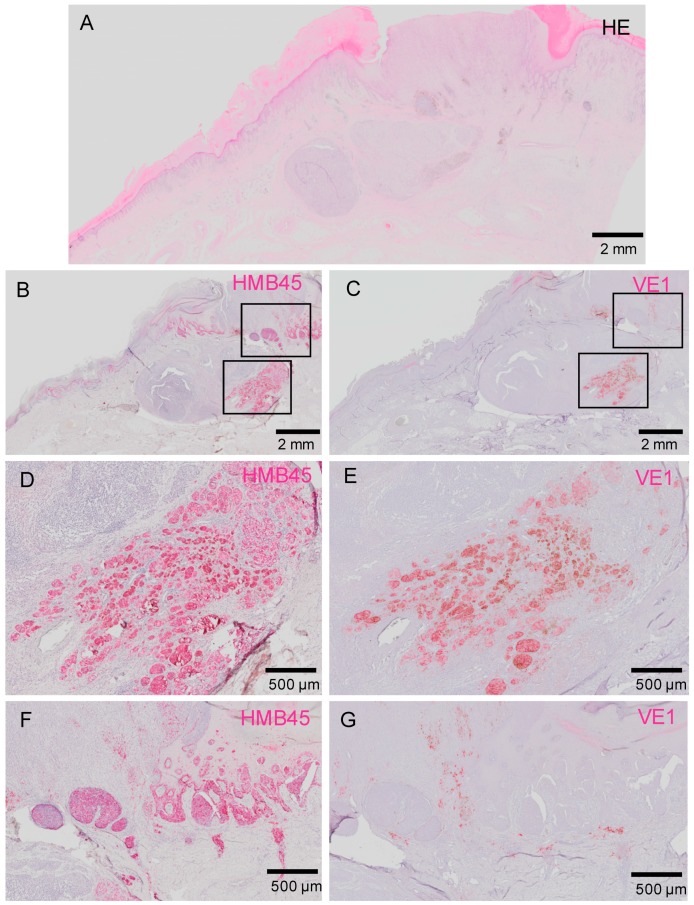
Another case of acral melanoma with marked heterogenous VE1 staining. Low-power views of the tumor (**A**–**C**). Proliferation of various subclones of melanoma cells with different VE1 positivity is obvious. (**D**,**E**) Only half of HMB45-positive melanoma cells show VE1 positivity. (**F**,**G**) None of the melanoma cells show VE1 positivity in this area. Bars indicate 2 mm in (**A**–**C**) and 500 μm in (**D**–**G**).

We found that some of the VE1-positive melanomas stained heterogeneously within tumors. Figure 3 shows melanoma that displays markedly heterogenous *BRAF*^V600E^ expression. This is a primary acral melanoma (histopathologically, an acral lentiginous melanoma) arising on the foot of a 65 years old male. This image clearly highlights the presence of melanoma subclones with different *BRAF*^V600E^ status. Another patient with heterogenous staining of VE1 is shown in Figure 4.

#### 2.3.2. Inter-Tumor Heterogeneity of *BRAF*^V600E^ in Acral Melanoma

**Figure 5:**
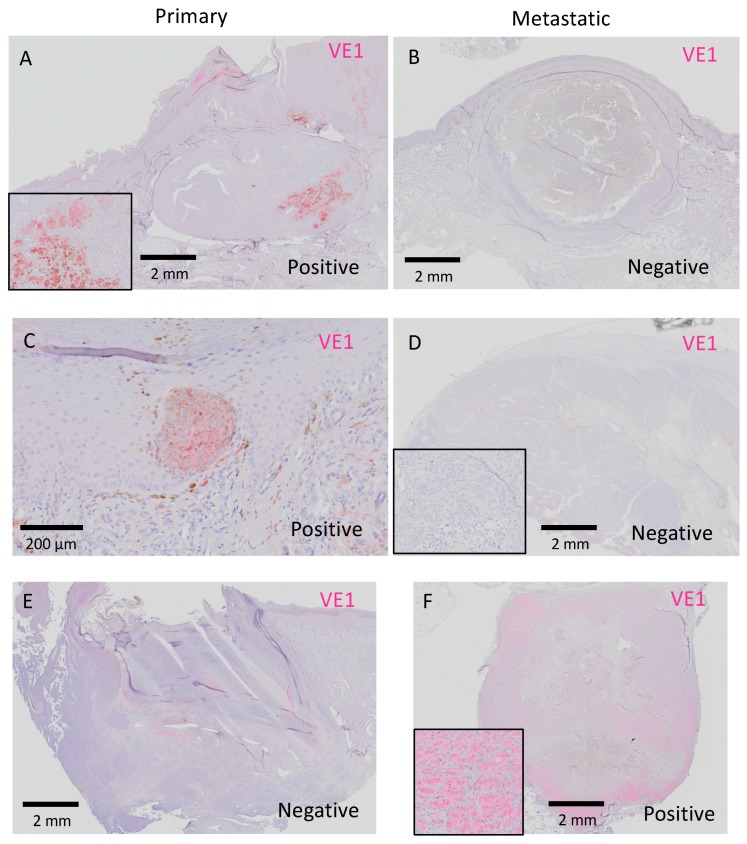
Matched pairs of primary and metastatic acral melanomas from three patients with discordant staining between primary and metastatic lesions. Black rectangles show representative high-power views of each figure (**A**,**D**,**F**). Bars indicate 2 mm in (**A**,**B**,**D**–**F**) and 200 μm in (**C**).

Next, we examined whether the *BRAF*^V600E^ status was discordant between primary and metastatic melanoma in the same patient (inter-tumor heterogeneity). Table 3 summarizes the results of VE1 staining in primary and metastatic lesions. Overall, nine primary (29.0%) and eight metastatic (25.8%) melanomas stained positive for VE1. Consistent mutation patterns in primary acral melanomas and matched metastases were observed in 28 of 31 patients (90.3%). Notably, the *BRAF*^V600E^ status in metastatic melanoma differed from that in primary melanoma in three patients (9.7%). Of the three patients, two patients had VE1-positive primary melanoma and VE1-negative metastatic melanoma, but an inverse staining pattern (negative in the primary and positive in the metastatic melanoma) was observed in one patient. VE1 staining of primary and metastatic melanomas in these discrepant patients are shown in Figure 5.

## 3. Discussion

Although the recent development of molecular targeted therapy and immunotherapy has opened a new era of melanoma treatment [24,25], it is still unclear if these therapies can be applied to acral melanoma, because the pathobiology and genetic heterogeneity of acral melanoma have not fully been elucidated due to the rarity of acral melanoma. To address these issues, we conducted a retrospective study on acral melanoma, finding several important issues. First, IHC using the VE1 antibody yielded results that were comparable with standard molecular analyses in detecting *BRAF*^V600E^ mutations. Second, clear proliferations of subclones with different *BRAF* status can exist within a tumor (intra-tumor heterogeneity) in patients who have acral melanoma. Third, some metastatic lesions of acral melanoma had a different *BRAF* status compared with primary lesions (inter-tumor heterogeneity).

Previous studies have revealed that IHC using the VE1 antibody can detect *BRAF*^V600E^ gene mutations with high sensitivity and specificity (both tests show a >95% concordance rate compared with molecular analysis) [12,18,26,27,28,29,30,31,32,33,34]. An IHC study that examined 171 melanoma samples from 81 patients found a high correlation between immunopositivity for the mutated protein and *BRAF*^V600E^ mutation detected by pyrosequencing [18]. Another study reported similar results from 140 patients in a comparison between IHC and RT-PCR, and the authors noted the high efficiency of the VE1 antibody for detecting *BRAF*^V600E^ [29]. A recent report from France dealing with 189 melanomas from 100 patients tested the sensitivity and specificity of the VE1 antibody compared with molecular testing. The high sensitivity (98.6%) and specificity (97.7%) of VE1 were confirmed in the study [27]. Fisher et al. provided results of their detailed examination of VE1 staining to determine when *BRAF* mutation should be regarded as positive in IHC [31]. The authors concluded that IHC using VE1 had good specificity and positive predictive value when stringent, consensus-scoring criteria were implemented, and the researchers proposed a cutoff value of >10% melanoma cells with more than moderate staining intensity (≥2+) [31]. In the current study, we defined a tumor as immunopositive when >5% of melanoma cells expressed the VE1 staining in accordance with the Cobas BRAF V600E Mutation Test. However, even if the cutoff value were set to 10% in our study, the results would not change.

It is sometimes difficult to differentiate the positive signals of IHC from melanin deposition when staining with a standard procedure using 3,3’-diaminobenzidine (DAB) as a chromogen, because the DAB and melanin have a similar brown color, causing possible misjudgment of IHC results. To avoid this kind of misjudgment, we stained slides with FastRed II, which reports positive signals in red and thus enabled us to clearly distinguish IHC staining from melanin [12,30].

Although the standard procedures for detecting *BRAF* mutations are DNA-based, IHC has many advantages: IHC requires less tumor tissue, detects *BRAF*^V600E^ mutations in specimens with low tumor content, and enables visualization of the location of mutated melanoma cells. IHC is also less expensive, produces more rapid results, consumes less formalin-fixed paraffin-embedded (FFPE) tissue, and is widely established as a routine technique in pathology departments. Therefore, IHC may be a useful ancillary method to detect *BRAF*^V600E^ mutations in melanoma. As far as we know, this is the first study to investigate acral melanoma *BRAF*^V600E^ mutations in detail using the VE1 IHC antibody. Only a few reports have investigated the *BRAF* status of acral melanoma in comparison with primary and metastatic lesions, and these studies were limited by small sample sizes (range of 4–16 patients) [26,28,35,36].

Our IHC studies clearly visualized the intra-tumor heterogeneity (heterogeneity within an individual melanoma). There have been debates regarding this issue, and conflicting results have been reported [15,27,28,29,30,33]. In our cohort, we found remarkable intra-tumor heterogeneity, as shown in Figure 3 and Figure 4. We cannot currently provide a definite hypothesis about the causes of conflicting reports regarding intra-tumor *BRAF* heterogeneity, but the fact that we exclusively dealt with acral melanoma might have contributed to the high rate of heterogeneity reported.

As for inter-tumor heterogeneity (heterogeneity among different melanoma sites in a patient), many researchers have mentioned the discrepancy of *BRAF* status between primary–metastatic and metastatic–metastatic melanoma [17]. Valachis et al. conducted a meta-analysis to solve this issue and concluded that a clinically meaningful discrepancy rate in *BRAF* status exists both between primary and metastatic tumors and among metastasis at different sites [17]. The pooled discrepancy rates were 13.4% (primary–metastatic) and 7.7% (metastatic–metastatic), respectively [17]. Our data showed similar results regarding the discrepancy rate (9.7%) between primary and metastatic acral melanomas. Potential *BRAF* inter-tumor heterogeneity is clinically important because of the risk of treating patients with unnecessary targeted therapies (loss of mutation in metastasis) or disturbing beneficial targeted therapies from patients (mutations in metastasis but not in primary melanoma). Taking these findings into account, we believe that repeated screening of *BRAF*^V600E^ status using VE1 antibody is important when a new metastatic melanoma occurs in patients with previously *BRAF* mutation-negative melanoma; this screening helps ensure that physicians do not overlook patients who are eligible for BRAF inhibitor treatment.

Besides the potential biases inherent in the retrospective design, one limitation of this study is that the VE1 antibody detects *BRAF*^V600E^ (both V600E1 and V600E2) but does not detect non-V600E *BRAF* mutations such as *BRAF*^V600K^ or *BRAF*^V600R^ [37,38,39]. Although the VE1 antibody shows high sensitivity and specificity for *BRAF*^V600E^, additional molecular testing is necessary to identify other *BRAF* mutations (V600K, V600D, V600R, and V600M, which together constitute <10% of *BRAF* mutations) when VE1 is negative.

In conclusion, we investigated *BRAF*^V600E^ mutation status in matched primary and metastatic acral melanoma patients. Some acral melanomas had marked intra-tumor heterogeneity. Approximately 10% of metastatic acral melanomas showed a *BRAF*^V600E^ mutation status that was different from the matched primary acral melanoma. IHC testing using the VE1 antibody is a useful ancillary method for the detection of *BRAF*^V600E^.

## 4. Materials and Methods

### 4.1. Ethics Statement

We conducted this investigation in accordance with the concepts enshrined in the Declaration of Helsinki. This study was approved by the Institutional Ethics Committee of Kyushu University (identification cord: 30-363, date: 27 November 2018).

### 4.2. Patients and Tissue Samples

We identified a total of 196 patients with primary cutaneous acral melanoma who were treated at the Department of Dermatology, Kyushu University, Fukuoka, Japan, between June 2001 and June 2019. All of the patients have agreed to participate in our study, and written informed consent has been obtained. For all the patients, at least three experienced dermatopathologists confirmed the diagnosis. Among these patients, both primary and metastatic acral melanoma tissues were available for 31 patients. All formalin-fixed (24 h in 10% buffered formalin) and paraffin-embedded (i.e., formalin-fixed paraffin-embedded) tissues were obtained from our hospital’s archives. Clinical and demographic data were retrieved from the patients’ files.

### 4.3. Immunohistochemistry

IHC staining was performed as reported previously [40,41,42,43]. The archival FFPE tissue blocks were cut into 4 μm thick tissue sections, deparaffinized, and rehydrated. Antigen retrieval was performed using Heat Processor Solution pH 9 (Nichirei Biosciences) at 100 °C for 45 min. Nonspecific binding was blocked using a supernatant of 5% skimmed milk. The sections were then incubated with mouse monoclonal antibody against human-mutated BRAF-V600E protein (VE1, ab228461, 1:100, Abcam) or a mouse monoclonal antibody against human HMB45 (HMB45, ENZ-30930, ready-to-use, Enzo, Enzo Life Sciences, NY, USA) at room temperature for 90 min, followed by incubation with an antibody, N-Histofine Simple Stain AP MULTI (Nichirei Biosciences), for 30 min. Immunoreactions were detected using FastRed II (Nichirei Biosciences) as a chromogen and were counterstained with hematoxylin. Sections stained without a primary antibody served as negative controls.

## Figures and Tables

**Table 1:** Clinicopathological data of all acral melanoma patients. (SD = standard deviation, PCR = polymerase chain reaction, IHC = immunohistochemistry).

Parameters	Number (%)
Age in years	
Range (mean ± SD)	32–88 (69.8 ± 12.3)
Sex	
Male	15 (48.4)
Female	16 (51.6)
Race/ethnicity	
Japanese	31 (100.0)
Type of melanoma	
Acral melanoma	31 (100.0)
Primary tumor site	
Hand	8 (25.8)
Foot	23 (74.2)
Site of metastasis	
Lymph node	24 (77.4)
Skin	6 (19.4)
Lung	1 (3.3)
Detection method for *BRAF*^V600E^	
IHC	21 (67.7)
IHC + real-time PCR	10 (32.3)
Total	31 (100)

**Table 2:** Correlation between real-time PCR and IHC in *BRAF*^V600E^ detection. (PCR = polymerase chain reaction, IHC = immunohistochemistry, 3+ = strongly positive staining, 2+ = moderately positive staining, 1+ = weakly positive staining, − = no staining).

Real-Time PCR	IHC (Proportion, Intensity)
Positive	Positive (100%, 3+)
Negative	Negative (3%, 3+)
Negative	Negative (2%, 2+)
Negative	Negative (3%, 1+)
Negative	Negative (0%, –)
Negative	Negative (0%, –)
Negative	Negative (0%, –)
Negative	Negative (0%, –)
Negative	Negative (0%, –)
Negative	Negative (0%, –)
Sensitivity, 100%; specificity, 100%	

**Table 3:** Discrepancy of *BRAF*^V600E^ status between primary and metastatic acral melanoma.

Mutation Status		*BRAF*^V600E^ Mutation	
	Primary Melanoma	Metastatic Melanoma	Number (%)
Concordance	Positive	Positive	7 (22.6)
	Negative	Negative	21 (67.7)
Discordance	Positive	Negative	2 (6.5)
	Negative	Positive	1 (3.2)

## Abbreviations

MAPK	mitogen-activated protein-kinase
RT-PCR	real-time PCR
IHC	immunohistochemistry
FFPE	formalin-fixed paraffin-embedded
